# Natural Products for the Treatment of Autoimmune Arthritis: Their Mechanisms of Action, Targeted Delivery, and Interplay with the Host Microbiome

**DOI:** 10.3390/ijms19092508

**Published:** 2018-08-24

**Authors:** Steven Dudics, David Langan, Rakeshchandra R. Meka, Shivaprasad H. Venkatesha, Brian M. Berman, Chun-Tao Che, Kamal D. Moudgil

**Affiliations:** 1 Baltimore Veterans Affairs Medical Center, Baltimore, MD 21201, USA; sdudics1@gmail.com (S.D.); david.langan@umaryland.edu (D.L.); Rmeka@som.umaryland.edu (R.R.M.); hvshivaprasad@gmail.com (S.H.V.); 2 Department of Microbiology and Immunology, University of Maryland School of Medicine, Baltimore, MD 21201, USA; 3 Family and Community Medicine, University of Maryland School of Medicine, Baltimore, MD 21201, USA; bberman@som.umaryland.edu; 4 Center for Integrative Medicine, University of Maryland School of Medicine, Baltimore, MD 21201, USA; 5 Medicinal Chemistry and Pharmacognosy, College of Pharmacy, University of Illinois at Chicago, Chicago, IL 60612, USA; chect@uic.edu; 6 Division of Rheumatology, Department of Medicine, University of Maryland School of Medicine, Baltimore, MD 21201, USA

**Keywords:** adjuvant-induced arthritis, arthritis, celastrol, curcumin, dietary supplements, EGCG, green tea, inflammation, liposomes, microbiome, nanoparticles, natural products, resveratrol, rheumatoid arthritis, targeted delivery, traditional medicine, Tripterygium wilfordii, triptolide

## Abstract

Rheumatoid arthritis (RA) is a chronic, debilitating illness characterized by painful swelling of the joints, inflammation of the synovial lining of the joints, and damage to cartilage and bone. Several anti-inflammatory and disease-modifying drugs are available for RA therapy. However, the prolonged use of these drugs is associated with severe side effects. Furthermore, these drugs are effective only in a proportion of RA patients. Hence, there is a need to search for new therapeutic agents that are effective yet safe. Interestingly, a variety of herbs and other natural products offer a vast resource for such anti-arthritic agents. We discuss here the basic features of RA pathogenesis; the commonly used animal models of RA; the mainstream drugs used for RA; the use of well-characterized natural products possessing anti-arthritic activity; the application of nanoparticles for efficient delivery of such products; and the interplay between dietary products and the host microbiome for maintenance of health and disease induction. We believe that with several advances in the past decade in the characterization and functional studies of natural products, the stage is set for widespread clinical testing and/or use of these products for the treatment of RA and other diseases.

## 1. Introduction

Rheumatoid arthritis (RA) is a multifactorial disease that involves both genetic predisposition and environmental components [1,2,3,4,5]. RA is prevalent worldwide with approximately 1.3 million people affected by RA in the United States alone [6,7,8,9,10]. Moreover, women are more likely to develop RA than men [7]. It is anticipated that with people living longer, the incidence of RA is likely to increase [11,12,13,14]. RA is typically characterized by chronic inflammation of the synovial membrane that lines the joints, damage to the cartilage, and erosion of the bone [15,16,17]. Swelling and redness of the hands and feet is the most common sign of RA along with pain in the afflicted areas [15]. Ulnar deviation [18], Swan neck deformity [19], and subcutaneous nodules [20,21] are among the clinical manifestations of untreated severe RA. The most common serum biomarkers for RA are rheumatoid factor (RF) and anti-citrullinated protein/peptide antibodies (ACPA) [22]. Furthermore, ACPA can also be used as prognostic markers for RA similarly to RF, as they are present a median of 4.5 years prior to clinical onset of the disease [23]. A relatively new potential biomarker for RA is the oncoprotein survivin [24], which is already a known biomarker for cancer. In one study, survivin was detected in 50.7% of RA patients but only 5.6% in controls, which indicates its high specificity [24].

## 2. The Cellular and Soluble Mediators of Arthritic Inflammation

**Figure 1:**
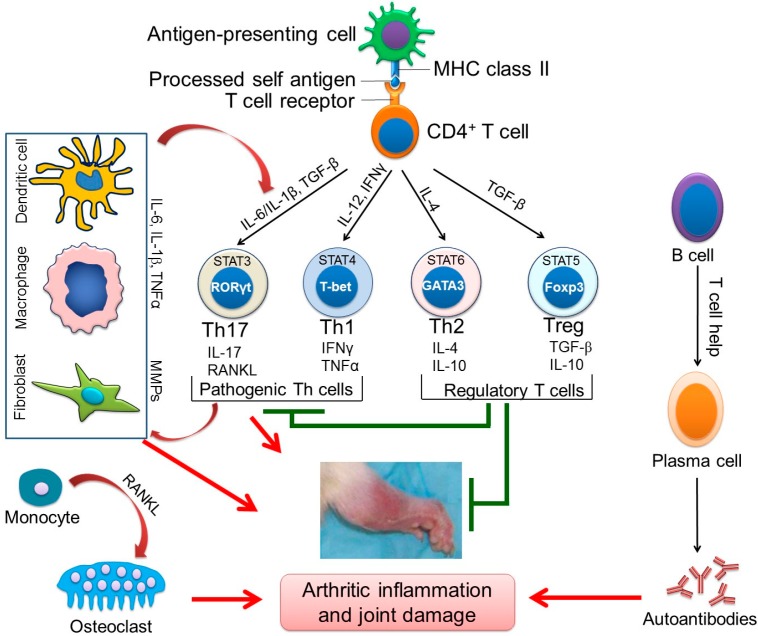
Immunopathogenesis of experimental autoimmune arthritis. Schematic representation of the key pathways is shown: The presentation of an autoantigen to autoreactive T cells and their differentiation into major T helper (Th) cell subsets under the influence of various cytokines; the activation and secretion of pro-inflammatory cytokines by myeloid cells; the T-B cell collaboration leading to autoantibody production by plasma cells; and the osteoimmune cross-talk leading to osteoclast differentiation. These intricate pathways regulate autoimmune inflammation of the synovial joint as shown by arrows (leading to activation/induction) and blunt ends (leading to suppression/inhibition).

Under normal conditions, the mature T cells encounter self-antigens in the periphery all the time; however, their activation is kept under control via diverse mechanisms, including unresponsiveness due to lack of adequate interaction between the peptide-MHC (major histocompatibility complex) complex and the T cell receptor (TCR) (ignorance), induction of anergy in the absence of co-stimulation, or suppression by T regulatory (Treg) cells [25,26]. The initiation of RA involves an interplay among components of the innate and adaptive immune responses leading to unintended activation of autoreactive T cells specific for potentially arthritogenic self-antigens in the peripheral lymphoid organs (Figure 1) [15,27]. Antigen-presenting cells (APCs), including dendritic cells, macrophages as well as activated B cells, present arthritogenic autoantigens to T cells that have specific TCRs that can recognize these autoantigens. At the same time, upregulation of co-stimulatory molecules expressed by the APCs under inflammatory conditions facilitates activation of these potentially arthritogenic T cells. Further, the cytokine milieu of the inflammatory environment (such as interleukin-12 (IL-12) and interferon-γ (IFN-γ) for T helper 1 (Th1), and IL6 and IL-1β for Th17) facilitates the differentiation of activated T cells into pathogenic T cell subsets (Th1, Th17), the key drivers of RA pathology [28,29]. This process results in break in tolerance leading to expansion of pathogenic T cells in the peripheral lymphoid organs.

The development of inflammatory arthritis in the joints involves the migration of activated pathogenic T cells from the peripheral lymphoid tissues into the joint tissue (synovial tissue), which is mediated primarily by a chemotactic process [15,27]. These T cells initiate joint-destructive activities by secreting cytokines and other mediators described below. This creates an inflammatory environment which attracts other cell types such as neutrophils, macrophages and fibroblasts to the local site. Collectively, these cells together with various effector molecules (e.g., cytokines, prostaglandins, proteolytic enzymes and other osteoclastogenic factors) induce joint inflammation and cartilage and bone damage. The B cells contribute to the pathogenesis of RA, not only through antigen presentation to the T cells, but also through the production of cytokines and autoantibodies, such as RF and ACPA, which can further reinforce the inflammation induced by the T cells [15,27,30,31]. Similarly, the Th17 cells produce receptor activator of nuclear factor kappa-Β ligand (RANKL), which along with other soluble mediators produced by myeloid cells, facilitates osteoclastogenesis. These osteoclasts can cause bone damage via secreting matrix-degrading enzymes such as matrix metalloproteinases (MMPs) and cathepsin K.

RA is associated with increased production of citrullinated proteins/peptides, which is usually attributed to smoke inhalation. Smoking causes irritation of the lungs and airways, in turn leading to an increase in peptidyl arginine deiminase (PAD) 2 [3], which catalyzes the conversion of arginine to citrulline in certain proteins. This increase in citrullinated proteins/peptides can cause an upregulation of antibodies to citrullinated protein/peptide antigens (ACPA), and individuals with certain human leukocyte antigen (HLA) alleles (e.g., HLA-DRB1) are more prone to mount an immune response to these modified antigens than others [4,5].

## 3. Animal Models of RA for Studying Disease Pathogenesis and Testing Therapeutic Agents

There are several different animal models used to study the disease process and test novel therapeutics for RA. One of the most well-known models is the collagen-induced arthritis (CIA) model. Here, DBA/1 mice are immunized with type II collagen (CII) emulsified in complete Freund’s adjuvant (CFA). This initiates both a B and T cell response to CII, leading to arthritis induction [32,33]. Another commonly used model is the adjuvant-induced arthritis (AA) model [34,35]. In AA, Lewis rats are immunized with heat-killed *Mycobacterium tuberculosis* H_37_R_a_ (Mtb) emulsified in mineral oil. This immunization activates a T cell response to mycobacterial heat-shock protein 65 (Bhsp65), which in turn leads to destruction of the joints [33,36,37,38]. A relatively newer transgenic mouse model to study RA is the K/BxN mouse [39], which were generated by crossing the TCR-transgenic KRN mice with non-obese diabetic (NOD) mice expressing the MHC class II molecule I-A^g7^. The K/BxN mice develop a severe and destructive form of arthritis, which is associated with high titers of antibodies recognizing glucose-6-phosphate isomerase (GPI), making it a valuable serum transfer model of arthritis [33,40]. Several researchers also use the proteoglycan-induced arthritis (PGIA) model in BALB/c mice, which is a T cell–dependent and autoantibody/B cell–driven disease [41]. Humanized mice are also used to study arthritis [2,33,42]. These mice express the human form of RA-susceptible MHC, HLA-DR4 and/or HLA-DQ8. This allows the researchers to study arthritis in mice but correlate the pathology to humans due to the human genetic background [2,33,42].

## 4. Currently Used Drugs for Arthritis Therapy and Their Limitations

There are multiple drugs currently being utilized to treat RA patients. The most widely used class of drugs is the non-steroidal anti-inflammatory drugs (NSAIDs) [43]. There are several different drugs under this category, including Ibuprofen, Aspirin, and Naproxone. A majority of them target and suppress prostaglandins (PGs) through inhibition of the cyclooxygenase (COX) enzymes [43]. Patients taking NSAIDs may experience a wide variety of symptoms including renal, hepatic, and cardiovascular toxicity [43]. Certain COX-2-selective agents can cause myocardial infarction (heart attack) and stroke [43]. The second major class of antirheumatic drugs is the disease-modifying antirheumatic drugs (DMARDs), and they belong to two categories, chemical and biologic [44]. Methotrexate (MTX) is one of the gold standards of therapy for RA, and it is a chemical DMARD. There is some evidence that MTX can suppress the production of proinflammatory cytokines [45,46] as well as modulate the levels of specific MMPs [47,48]. Another proposition that has been corroborated in animal models and humans is the augmentation of adenosine levels through the reduction of adenosine deaminase in lymphocytes [49]. Adenosine exerts its anti-inflammatory properties via inhibiting proinflammatroy cytokine production, attenuating neutrophil trafficking to sites of inflammation, and suppressing Th17 cell differentiation, while stimulating Treg differentiation [50]. Adenosine binds to the A_2A_ receptor, which is increased on the surface of lymphocytes in RA patients, and exert its immunosuppressive properties [50]. While the MTX response rate in RA patients is approximately 50% [51], the long-term use of this drug can lead to liver fibrosis, which in some cases may require a liver transplant for its management [52,53,54].

The biologic DMARDs include monoclonal antibodies targeting tumor necrosis factor (TNF)-α (anti-TNF-α) and IL-6 receptor (anti-IL6R), thereby inhibiting these two cytokines that are major players in promoting RA pathogenesis. Anti-TNF-α is currently the standard of care for RA patients, and it is widely used either alone or in combination with other drugs such as MTX [55,56]. Approximately 10–30% of patients do not respond to initial treatment with anti-TNF-α, and another 23–46% lose responsiveness over time [57]. Moreover, due to the immunosuppressive effect of blocking TNF-α, RA patients are at an increased risk of recurrent infections [58]. Anti-IL6R has been shown to be efficacious in suppressing RA [59,60], and it can be useful in patients not responding to other forms of treatment [61]. However, anti-IL6R has similar side-effects as anti-TNF-α [62].

Corticosteroids are another group of biologic DMARDs, which has been used for RA therapy for the past several decades [63]. Corticosteroids suppress inflammation by binding to the glucocorticoid receptor (GR), also known as NR3C1 (nuclear receptor subfamily 3 group C member 1), in turn leading to the transcription of multiple genes that inhibit several inflammatory pathways. These genes include glucocorticoid-induced leucine zipper protein (GILZ) and MAP kinase phosphatase-1 (MKP-1), which suppress the nuclear factor kappa light chain enhancer of activated B cells (NF-κB) (and Activator protein 1 (AP-1)) and p38 MAP kinase, respectively [64]. Moreover, corticosteroids can lead to an epigenetic modification of histones, subsequently resulting in reduction in inflammation [64]. Corticosteroids have unwanted side effects, including osteoporosis, peptic ulcer, and increased rate of infections [65,66].

## 5. The Use of Plant Natural Products for Arthritis Therapy

While the above-mentioned mainstream drugs are widely prescribed to patients, they have unwanted side effects. Moreover, some of these drugs are quite expensive. Owing to these limitations, an increasing number of patients have started to turn to natural products to relieve symptoms of RA and related ailments [67], with over 36% of adults in the USA using complementary and alternative (CAM) therapies [68]. Natural products have been studied extensively in multiple different ailments such as cancer [69], infectious diseases [70], and autoimmunity [71,72,73,74,75]. However, difficulties in evaluating the efficacy of these products as well as inadequate information about their mechanism of action are among the reasons for skepticism from both the public and professional communities [76,77]. Therefore, defining the mechanism of action of natural products is a high priority, as also emphasized by National Center for Complementary and Integrative Health (NCCIH)/National Institutes of Health (NIH), USA. A summary of significant validation of some of the common natural products in arthritis therapy is given below.

Natural products can control arthritic inflammation through multiple pathways, for example, inhibition of effector molecules (e.g., pro-inflammatory cytokines and chemokines), induction of anti-inflammatory mediators (e.g., IL-4, IL-10), regulation of the Th17/Treg balance, and modulation of the osteo-immune cross-talk [34,78,79,80,81]. These effects are in turn the outcome of the control of molecular mediators of inflammation such as NF-κB (nuclear factor kappa-light-chain-enhancer of activated B cells), MAPK (mitogen-activated protein kinase), and STAT3 (signal transducer and activator of transcription 3) by the bioactive components of plant-derived or other natural products. Furthermore, natural products can modulate the Th17/Treg balance by controlling the relative levels of key cytokines (e.g., IL-1β, IL-6, and TGF-β (transforming growth factor-β)) and certain transcription factors such as STAT3, RORγt (RAR-related orphan receptor gamma), IRF-4 (interferon Regulatory Factor 4), and Foxp3 (forkhead box P3) [34,82]. Similarly, acting via certain cytokines (e.g., IL-17) and other mediators such as RANKL, natural products can influence not only the T cell response, but also the osteo-immune cross-talk and bone health [80,83,84,85]. In this regard, natural products possessing the above properties can serve as potential therapeutic agents to treat RA either alone or in combination with certain mainstream anti-arthritic drugs. However, such combination therapies need to be fully assessed for their compatibility in regard to any unexpected drug interactions that might affect efficacy and side effects of these therapeutic agents.

We have discussed below in more detail some of the well-studied natural products for arthritis therapy. Most of the information is derived from testing in animal models, but where applicable, results of clinical testing in patients are also summarized.

### 5.1. Tripterygium wilfordii Hook F

#### 5.1.1. Tripterygium wilfordii Hook F (TwHF)

One of the most well-studied natural products for its therapeutic properties in RA, TwHF has the capabilities to suppress numerous pro-inflammatory mediators. For example, TwHF extract reduces in vitro production of prostaglandin E2 (PGE2) through the suppression of COX-2 [86,87]. In addition, TwHF inhibits the production of nitric oxide (NO) by targeting and attenuating the transcription of the inducible nitric oxide synthase (iNOS) gene [86,88]. Moreover, in human synovial fibroblasts, TwHF reduces the expression of MMP1 and MMP3, while augmenting tissue inhibitors of metalloproteinases (TIMPs) that inhibit MMP1 and MMP2 [86,89]. Furthermore, TwHF blocks the transcription of MMP3 and MMP13, thereby reducing their levels [86,90]. The transcription of iNOS and COX2 is under the control of NF-κB, and TwHF has been shown to prevent NF-κB from binding to DNA, inhibiting its activation [86,90]. In turn, this leads to reduced levels of NO and COX2. Furthermore, TwHF can suppress the production of numerous proinflammatory cytokines (e.g., TNF-α, IL-1β, IL-6, IL-8, and IFN-γ) from T cells and macrophages. The balance of the T cell subsets and their differentiation is crucial for the development and propagation of RA [91]. It has been shown that TwHF can suppress T/B cell proliferation and synovial fibroblast growth [92,93,94] as well as induce T cell apoptosis [95].

Finally, the testing of TwHF in RA patients showed that it is an efficacious form of therapy for RA both alone and in combination with conventional (mainstream) therapies such as MTX, sulfasalazine (a chemical DMARD), or other DMARDs [96,97,98,99,100,101,102,103]. These included randomized, placebo controlled trials as well. One study used local application of TwHF formulation, but the rest used oral administration of TwHF. Different treatment regimen used in these studies can be summarized as follows: (a) RA patients treated with TwHF alone compared with control group that received placebo, or not [97]; (b) RA patients treated with TwHF compared with those treated with a DMARD (either MTX or sulfasalazine) [96,99,100,103]; and (c) RA patients treated with a combination of TwHF and MTX compared with control group that received MTX alone [98,101,102]. Taken together, the results of these studies, which also included meta-analysis, showed that TwHF alone was just as effective as either MTX or sulfasalazine alone. However, the most efficacious outcome was with a combination of TwHF with MTX. The safety profile of TwHF alone or in combination with a DMARD was comparable to that of DMARD alone. While TwHF is generally safe for human consumption, patients in these trials reported some side effects, the most common being gastrointestinal distress [103].

#### 5.1.2. Triptolide

A bioactive component of TwHF, triptolide can induce T cell apoptosis and inhibit the phosphorylation of STAT3 [104,105]. In addition, triptolide can attenuate the NF-κB activity and several other pathways induced by TNF and TLR4 in RA [106,107,108]. Also, in CIA, triptolide can protect against bone damage by targeting RANKL-mediated ERK/Akt signaling [106,108]. Triptolide can also target and suppress the IL-6/STAT3/SOCS3 pathway in lamina propria mononuclear cells and promote their apoptosis [106,109], as well as attenuate angiogenesis [110].

#### 5.1.3. Celastrol

Another bioactive component of TwHF, celastrus, and some other members of celastraceae family, celastrol has multiple anti-arthritic properties. Studies from our laboratory [34,79,80,84,111] and others [78,111] have demonstrated that celastrol can reduce arthritis severity in the AA model in Lewis rats [84]. Moreover, celastrol can suppress proinflammatory cytokine production as well as skew the T cell balance to a regulatory phenotype in the target organ, the joints [34,79,84]. In addition, celastrol can protect against bone erosion by targeting the RANKL pathway and deviating the RANKL/osteoprotegerin ratio that aids in the inhibition of osteoclastic activity. In turn, this reduces the osteoclast numbers and subsequent bone damage [80].

### 5.2. Green Tea

Green tea (*Camellia sinensis*) has several anti-inflammatory properties [112,113]. It has been demonstrated that feeding green tea polyphenols to CIA mice prevented the onset and progression of arthritis [114]. This was associated with marked reduction of COX-2, IFN-γ and TNF-α in the joints, and of antibodies to CII in serum and arthritic joints. Similarly, feeding the green tea polyphenols to AA rats significantly reduced arthritic scores [112]. Moreover, these rats had reduced IL-17 levels and increased IL-10 levels in the draining lymph node cells (LNCs). Also, serum analysis showed a significant reduction in anti-Bhsp65 antibodies compared to controls [112]. Another study in the same model revealed that green tea had superior anti-arthritic capabilities compared to black tea. Here, rats treated with green tea had reduced arthritic scores compared to both black tea-treated and non-treated controls [113]. Proinflammatory cytokine (TNF-α and IL-1β) levels in the serum were significantly reduced in the rats given green tea compared to non-treated controls and black tea-treated rats [113]. Also, protein levels of C–C chemokine receptor type 5 (CCR5) in the joint were decreased in rats given green tea [113].

One of the main bioactive molecules of green tea is epigallocatechin-3-gallate (EGCG) [115], and it has been shown to target multiple inflammatory pathways. Using the rat AA model, it was shown that EGCG can inhibit arthritis, which was associated with inhibition of IL-6 synthesis as well as trans-signaling [116]. The latter effect was attributable to increased synthesis of soluble gp130. EGCG and other catechins in green tea can interfere with IL-1β signaling and reduce the levels of pro-inflammatory mediators IL-8 and IL-1β in synovial fibroblasts [117,118]. Further analysis revealed that EGCG was able to effectively target and inhibit transforming growth factor beta-activated kinase 1 (TAK-1) activity, subsequently reducing IL-8, IL-1β as well as MMP2 and COX2 levels [117,118]. Moreover, EGCG was able to suppress p38 and NF-κB activity more effectively than the other catechins [117]. Also, EGCG was shown to increase Nrf2 (nuclear factor, erythroid 2-like 2) and HO-1 (heme oxygenase-1) activities, subsequently upregulating the production of indoleamine-2,3-dioxygenase (IDO). IDO is able to suppress the differentiation of pathogenic T cell subsets, while inducing Treg differentiation [119]. Another study demonstrated that EGCG can also regulate and suppress Th17 cell production and inhibit osteoclastogenesis by inhibiting STAT3 signaling [120]. Moreover, MMP2 and MMP9 levels were reduced in osteoclasts that were treated with EGCG compared to non-treated controls [121], which further shows the ability of EGCG to inhibit bone erosion. Finally, there have been multiple studies detailing the effects of green tea consumption in RA patients and reduction in disease activity score-28 (DAS28) as well as better pain management. Moreover, green tea was shown to prevent RA more effectively when compared with high fat beverages [122].

### 5.3. Curcumin

Curcumin possesses a wide variety of anti-inflammatory properties which make it a potent anti-arthritic bioactive molecule. Curcumin is derived from turmeric (*Curcuma longa*), a plant product that has been shown to attenuate inflammation [123,124,125]. A recent study using the AA model in Lewis rats demonstrated that curcumin was as effective at treating arthritis as MTX [126]. Rats treated with curcumin showed attenuated disease and histopathology scores, as well as reduced levels of pro-inflammatory cytokines (TNF-α and IL-1β) in the serum and synovial fluid compared to controls [126]. Curcumin inhibits NF-κB activity, which, in turn, leads to the reduction in proinflammatory cytokines as well as prevents TNF-α-induced adhesion of monocytes to endothelial cells [73,127,128]. Curcumin also regulates the cyclooxygenase (COX) and lipoxygenase (LOX) enzymes, leading to the suppression of various proinflammatory mediators, including MMP9 and MMP13 [73,129,130,131,132], and subsequent inflammation [73,133]. Furthermore, curcumin has been shown to suppress IL-1β signaling in articular chondrocytes [134] through the downregulation of mitogen-activated protein kinase (MAPK), activator protein 1 (AP-1), and NF-κB pathways. In turn, this leads to the reduction of MMP9 and MMP13 production [134]. Finally, curcumin can reduce the osteoclastogenic potential of peripheral blood mononuclear cells (PBMCs) of RA patients [135]. This was evident from the inhibition of expression of receptor activator of nuclear factor κB (RANK), c-Fos and nuclear factor of activated T cells (NFATc1) following curcumin treatment [135]. Also suppressed were extracellular signal-regulated kinases 1 (ERK1) and ERK2, p38 and c-Jun N-terminal kinase (JNK) [135].

### 5.4. Resveratrol

Resveratrol, a polyphenol, is found in red grape and other plant sources [136]. Resveratrol can suppress arthritis in CIA mice [137,138]. The T cell profile of these mice showed a reduction in the number of Th17 cells in the draining lymph node as well as decreased levels of circulating pro-inflammatory cytokines, such as IL-17, and CII-specific antibodies [137,138]. Resveratrol also has osteoprotective properties [139,140]. CIA mice treated with resveratrol showed a reduction in bone erosion as well as cartilage damage as evidenced by Safranin-O and tartrate-resistant acid phosphatase (TRAP) staining, in addition to radiographic imaging of the paws [140]. Moreover, resveratrol treatment leads to the suppression of wingless-related integration site (Wnt) signaling, an important pathway for bone remodeling and synovial inflammation [141], by targeting Wnt5a and inhibiting its expression [139]. Intra-articular injection of resveratrol suppressed signs of inflammatory arthritis in rabbits as evidenced by reduced histological scores [142]. Resveratrol is able to modulate the MEK-ERK1/2, MAPK, AP-1 and NF-κB pathways in various tissues [143]. Furthermore, resveratrol plays a role in regulating the aryl hydrocarbon receptor (AhR), which is known to affect several immune-mediate pathways in RA [143,144,145,146]. A previous study has indicated that resveratrol can ameliorate arthritis and synovial hyperplasia by inhibiting angiogenesis [147]. Resveratrol-treated rats showed significantly attenuated articular cartilage degradation, and higher caspase-3 levels in inflammatory cells compared to non-treated controls [147].

### 5.5. Other Natural Products

There are several other natural products besides those described above that possess anti-inflammatory activities and are reported to be beneficial in the treatment of arthritis and some other musculoskeletal disorders in patients and experimental models [75]. Some of these are summarized in Table 1.

## 6. Nanoparticle-Based Delivery of Plant Natural Products and Other Drugs for Arthritis Therapy

**Figure 2:**
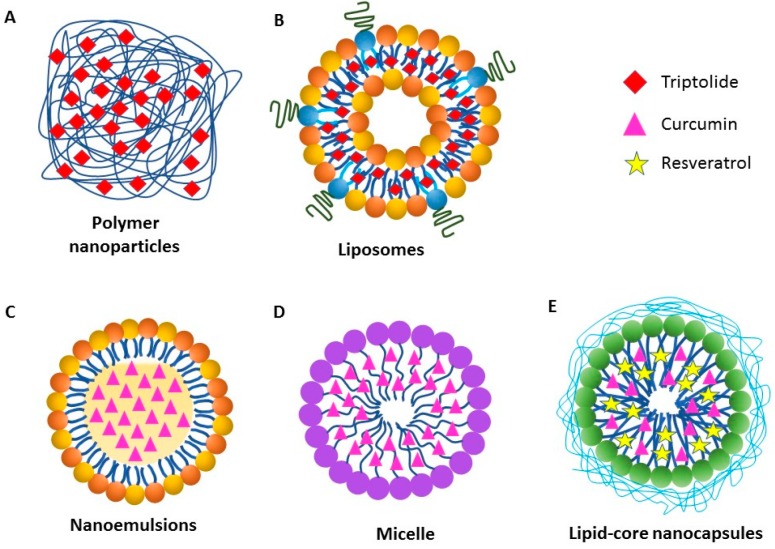
Schematic representation of different types of nanoparticles for the delivery of natural products. (**A**) Polymer nanoparticles encapsulating triptolide; (**B**) Liposomes loaded with triptolide; (**C**) Nanoemulsions entrapping curcumin; (**D**) Micelles encapsulating curcumin; and (**E**) Lipid core nanocapsules co-encapsulating resveratrol and curcumin.

A major challenge in the treatment of diseases using natural products is their poor absorption. This results in loss of bioavailability and efficacy, and it requires consumption of a large amount of the natural product to achieve the required therapeutic effects. This increase in dosage can lead to unwanted toxicity [162,163,164]. Therefore, novel methods for delivering natural products are needed to improve therapeutic efficacy as well as reduce toxicity. Nanotechnology is an attractive approach in this regard [165,166]. Currently, there are many types of nanoparticles that have been approved for clinical use, and these nanoparticles are being used for therapeutic and diagnostic purposes (Figure 2) [167,168]. Encapsulating the bioactive natural products into nanoparticles can increase their in vivo stability, extend their circulation time, and allow for their controlled and sustained release [169,170]. Furthermore, with suitable modifications, nanoparticles can deliver drugs to a particular target site, including inflamed tissues [171,172,173,174]. For this purpose the surface of the nanoparticles can be modified with a peptide, an antibody, a protein, or a small molecule [172] to direct them to the desired inflamed tissue or organ [170,175,176]. The most common types of nanoparticles used for drug delivery for therapeutic purpose are micelles, lipid nanoparticles, liposomes, polymeric nanoparticles and emulsions [177].

### 6.1. Polymer Nanoparticles

Polymer Nanoparticles are biodegradable, biocompatible, minimally immunogenic, and suitable for targeted delivery of therapeutic drugs [178]. For example, triptolide-loaded PGA (poly-γ-glutamic acid) polymer nanoparticles (Figure 2A) were found to be more safe and efficacious compared to free triptolide, when tested at similar dosage [179]. In that study, mice treated intravenously (i.v.) with nanoparticle -triptolide showed increased survival rates and significantly reduced liver and kidney toxicity, when compared with free triptolide-treated mice [179]. In another study, TNF-α transgenic mice treated with nanoparticle-triptolide had a survival rate of 100% and no body weight changes compared to the survival rate of 70% and 20% body weight loss, respectively in mice treated with free triptolide [180]. However, the difference between the two groups in the bone volume of astragalus (ankle bone) was not significant [180].

### 6.2. Liposomes

Liposomes are spherical-shaped nanovesicles that are extensively used as carriers for the delivery of therapeutic drugs [181]. Liposomes can encapsulate both hydrophobic and hydrophilic drugs and can release the entrapped drug at designated targets [182,183]. A study using triptolide-entrapped liposomes (Figure 2B) in a rat model of CIA [184] showed slower release and a longer half-life in plasma as well as decreased hepatic and digestive tract toxicity when compared to free triptolide. Furthermore, there was a decrease in IL-1β and IL-6 levels in serum as well as reduced expression of Flk-1, Flt-4, and HIF-1α in synovium of liposome-triptolide group when compared to arthritic control group [184]. In a recent study, we have shown that liposomes encapsulating an immunomodulatory cytokine IL-27 and displaying a joint-homing peptide [185] on their surface are more effective in suppressing AA in rats compared with liposomes containing IL-27 but lacking the peptide on the cell surface as well as free IL-27 [186]. We propose that IL-27 combined with a natural product such as celastrol [186] might have an additive or synergistic protective effect against AA, and we plan to test this in the near future.

### 6.3. Nanoemulsions

Nanoemulsions result from the dispersion of two immiscible liquids, typically water and oil, and are stabilized using an appropriate surfactant [187]. In a study on AA [126], free curcumin was administrated i.v., whereas the curcumin-nanoemulsion formulation (Figure 2C) was administrated orally. The plasma concentration of curcumin was increased three-fold, whereas the levels of TNF-α and IL-1β in both synovial fluid and serum were reduced two-fold in the curcumin-nanoemulsion-treated group compared to free curcumin-treated group [126].

### 6.4. Nanomicelles

Nanomicelles are self-assembling colloidal constructs composed of amphiphilic monomers (Figure 2D). Their hydrophobic core can encapsulate hydrophobic drugs/natural products, whereas the hydrophilic shell helps enhance the solubility of the drug. The surface of the nanomicelles is suitable for conjugation with cell/tissue-targeting ligands [188,189]. Nanomicelles were shown to improve the therapeutic efficacy of curcumin in CIA [190]. Curcumin-nanomicelles caused a significant reduction in paw edema, whereas free curcumin failed to reduce the paw swelling. Moreover, serum levels of IL-1β and vascular endothelial growth factor (VEGF) were significantly decreased in curcumin-nanomicelles-treated rats compared to free curcumin-treated rats [190].

### 6.5. Lipid-Core Nanocapsules

Lipid-Core Nanocapsules are composed of triglycerides/grape seed oil and sorbitan monostearate surrounded by a polymeric wall, which protects both the nanoparticle as well as the drug from degradation, and it also helps control drug release (Figure 2E) [191,192]. In one study, resveratrol and curcumin were co-encapsulated in lipid-core nanocapsules [137], and then administered intraperitoneally (i.p.) to arthritic rats, followed by monitoring of arthritis severity. These nanocapsules improved the edema-reducing activity of resveratrol and curcumin compared to their free counterparts, and no toxic side-effects were reported.

## 7. Interplay between Dietary Products and the Host Microbiome for Maintenance of Health and Disease Modulation

The human microbiota is composed of bacteria, fungi, and viruses, whose total population exceeds that of the host cells, and whose metabolic contribution to our state of health is essential [193,194,195]. From here on, the prefix ‘micro’ will be used to refer exclusively to prokaryotic organisms. The microbiome is an untapped source for identification of novel natural products for the treatment and prevention of disease such as RA. The associations between certain taxa of microbes and RA have been reported [196,197]. In addition, a variety of bioactive molecules produced by the human microbiome can have significant effects on the immune system [198]. There are three general approaches to harnessing the microbiome for RA prevention and treatment, namely the use of prebiotics, probiotics, and microbial-derived xenobiotics. Prebiotics are certain dietary ingredients metabolized by the microbiome to facilitate growth of beneficial bacteria in the gut, which in turn produce substances that are optimal for the local microenvironment and health of the intestines. Probiotics consist of living strains of beneficial bacteria. Microbial-derived xenobiotics are the molecules produced by the action of the microbiome action, for example, metabolic degradation/alteration of the dietary products consumed by the host and/or of the products released from the host’s cellular processes.

The best known examples of prebiotics are plant-derived fibers inulin and oligofructose. These are present in several grains (e.g., wheat), fruits (e.g., bananas), and vegetables (e.g., onion and garlic). These prebiotic fibers serve as a fuel for microbiota, which then produce substances that contribute to the optimal health and function of intestinal cells. Another way for microbiota to influence immune system is via production of xenobiotic metabolites. For example, *Bifidobacterium* spp., including *B. bifidum*, convert dietary carbohydrates into butyrate. In addition to propionate and acetate, these essential short-chain fatty acids (SCFAs) affect host cells by inhibiting histone-deacetylase to promote anti-inflammatory responses [198]. Oral administration of SCFAs prior to induction of CIA has been shown to reduce arthritis severity [199]. SCFAs were shown to inhibit osteoclastogenesis in vitro, and to prevent bone loss in the CIA and K/BxN models of arthritis when administered orally [200]. These studies illustrate the potential benefits of xenobiotic supplementation. It also enforces the importance of a complex carbohydrate rich diet. In addition to SCFAs, unexplored microbial-derived ligands for the AhR could prove efficacious for RA treatment, as other ligands of the AhR are either currently licensed for RA treatment or have been shown to alter disease in animal models [145,201]. In the dairy-derived *P. freudenreichii*, beneficial metabolites produced could include SCFAs as well as the metabolite 1,4-Dihydroxy-2-naphthoic acid (DHNA), a known AhR ligand.

## 8. Concluding Remarks

While current therapies are effective at mitigating RA, their prolonged use is associated with unwanted side effects. It is clear that natural products can be effective forms of therapy for RA. We described in detail four such natural products and their bioactive components, but there are many more that have been shown to possess anti-inflammatory and anti-arthritic properties. One of the major hurdles of using natural products for therapy is their poor bioavailability. In order to combat this issue, researchers are turning to nanoparticle delivery of such products, and have reported successful application of these approaches. Nanoparticles are designed for the delivery of drugs/biologics to improve their pharmacological and therapeutic properties. They can protect the drug against degradation and deliver the drug to a specific target. In consequence, lower dose of the drug is required to achieve the desired efficacy. Thus, nanoparticles can ensure controlled release of drugs and reduce their toxicity. Furthermore, natural products might also contain prebiotic components, whose interaction with the host microbiome can have a significant impact on health and disease. This is a new area of research that would further help optimize the selection of natural products for therapy and define their mechanisms of action. Taken together, in the past couple decades, there has been a gradual increase in the use of natural products for the maintenance of health and treatment of arthritis and other diseases all over the world. It is hoped that in the near future, with additional research efforts and controlled clinical testing, natural products will be more widely accepted and used as therapeutics, either alone or as adjuncts to mainstream allopathic (Western) drugs, for RA and other diseases.

## Figures and Tables

**Table 1:** Natural products with anti-inflammatory/anti-arthritic activity.

Plant Name	Bioactive Compounds	Mediators of Inflammation Targeted by the Natural Product	References
*Boswellia serrata*	Boswellic acids	Proinflammatory cytokines, 5-LOX	[148,149]
*Harpagophytum procumbens*	Harpagoside, Harpagide	COX-2, MAPK, NF-κB, NO	[150,151]
*Rosa canina*	Carotenoids, organic acids	COX-1, COX-2	[152,153]
*Uncaria tomentosa*	Mitraphylline	Pro-inflammatory cytokines, anti-oxidant, NF-κB	[154,155]
*Urtica dioica*	Flavonoid glycosides, terpenoids	NF-κB, PLA2	[156,157]
*Zingiber officinale*	Zingerone, Gingerol	COX-2, NF-κB	[158,159]
Several grains, vegetables, fruits	Quercetin	MAPK, NF-κB, PI3K/Akt, JAK3	[160,161]

LOX, lipoxygenase; NF-κB, nuclear factor-kappa B; PLA2, phospholipase A2; COX; cyclooxygenase; MAPK, mitogen-activated protein kinase; NO, nitric oxide; PI3K, phosphatidylinositol-3-kinase; Akt, also known as PKB, protein kinase B; JAK, janus kinase.

## Declaration

The contents do not represent the views of the U.S. Department of Veterans Affairs or the United States Government.
